# First report of *Bitylenchus ventrosignatus* (Tobar Jiménez, 1969) Siddiqi, 1986 associated with wild grass in Botswana

**DOI:** 10.21307/jofnem-2021-037

**Published:** 2021-03-26

**Authors:** Ebrahim Shokoohi

**Affiliations:** Green Biotechnologies Research Centre of Excellence, University of Limpopo, Private Bag X1106, Sovenga 0727, South Africa

**Keywords:** Botswana, *Bitylenchus*, Morphology, Phylogeny, rDNA

## Abstract

During a survey on the biodiversity of plant-parasitic nematodes of natural areas in Botswana, *Bitylenchus ventrosignatus* was discovered around the rhizosphere of wild grass. The nematodes were extracted using the tray method and then fixed according to the available protocols. The morphological characters fit well with the description of *B. ventrosignatus*. In addition, molecular analysis using 18 S and 28 S rDNA indicated 98% (KJ461617) and 95% (KJ461567) similarity with the Spanish population of *B. ventrosignatus*. The phylogenetic analysis of 18 S and 28 S rDNA placed the examined population with other populations of *B. ventrosignatus* in a group with a posterior probability support value of 100. According to published literature, this is the first report of *B. ventrosignatus* from Botswana.

The genus *Bitylenchus* belongs to the family Dolichodoridae Chitwood in Chitwood, 1950. This genus has been synonymized with *Tylenchorhynchus* (Geraert, 2011). However, Handoo et al. (2014) and Hosseinvand et al. (2020) considered it as a valid taxon using molecular analysis. Siddiqi (2000) considered 29 valid species under the genus *Bitylenchus*. Members of *Bitylenchus* and *Tylenchorhynchus* differ in areolated outer bands of lateral fields, a large postanal intestinal sac containing intestinal granules and fasciculi, relatively more thickened cuticle at the female tail tip, and gubernaculum lacking a crest (Handoo et al., 2014). However, their ecological behavior and crop damage are not well understood. During a survey on nematodes of the natural areas of Botswana, *B. ventrosignatus* (Tobar Jiménez, 1969) Siddiqi, 1986 was recovered from a wild grass in Botswana. According to published literature, this is the first report of *B. ventrosignatus* from Botswana.

## Materials and methods

### Nematode extraction and processing

Rhizosphere soil samples were collected from the natural veld. Specimens were collected in the North-West District of Botswana (S 20° 8’ 24.882”, E 21° 12’ 45.475”) from the rhizosphere of wild grass. Nematode extraction was achieved using the Baermann (1917) funnel technique. Extracted individuals were fixed with a hot 4% formaldehyde solution (except those specimens used for molecular analyses) and transferred to anhydrous glycerine utilizing the method of De Grisse (1969) and mounted on permanent glass slides. The classification provided by Handoo et al. (2014) was used for the taxonomic study of *Bitylenchus*.

### Light microscopy (LM)

Measurements were taken of specimens mounted on permanent slides, and De Man’s (1880) indices were calculated. Drawings were made using a drawing tube (camera lucida) attached to a Leitz Laborlux S microscope (Leitz, Wetzlar, Germany). Pictures were taken with a Nikon Eclipse 80i light microscope provided with differential interference contrast optics (DIC) and a Nikon Digital Sight DS-U1 camera (Nikon, Tokyo, Japan). Micrographs were edited using Adobe® Photoshop® CS.

The terminology used for the morphology of stoma and spicules-gubernaculum follows the proposals by Baldwin et al. (2004) and Abolafia and Peña-Santiago (2017), respectively.

### DNA extraction, PCR, and phylogenetic analysis

DNA extraction was done using the Chelex method (Straube and Juen, 2013). Five specimens of each species were hand-picked with a fine tip needle and transferred to a 1.5 mL Eppendorf tube containing 20 μL double distilled water. The nematodes in the tube were crushed with the tip of a fine needle and vortexed. Thirty microliters of 5% Chelex® 50 and 2 µL of proteinase K were added to each of the microcentrifuge tubes that contained the crushed nematodes and mixed. These separate microcentrifuge tubes with the nematode lysate were incubated at 56°C for 2 h and then incubated at 95°C for 10 min to deactivate the proteinase K and finally spin for 2 min at 16,000 rpm (Shokoohi et al., 2020). The supernatant was then extracted from each of the tubes and stored at –20°C. Following this step, the forward and reverse primers, SSU F04 (5’–GCTTGTCTCAAAGATTAAGCC–3’) and SSU R26 (5’–CATTCTTGGCAAATGCTTTCG–3’) (Blaxter et al., 1998) for 18 S rDNA and D2A (5’–ACAAGTACCGTGAGGGAAAGTTG–3’), D3B (5’–TCGGAAGGAACCAGCTACTA–3’) (De Ley et al., 1999) for 28 S rDNA, were used in the PCR reactions for partial amplification of the 18 S rDNA, and 28 S rDNA regions. PCR was conducted with 8 μL of the DNA template, 12.5 μl of 2X PCR Master Mix Red (New England Biolabs; NEB), 1μL of each primer (10 pmol μL–1), and ddH2O for a final volume of 30 μL. The amplification was processed using an Eppendorf master cycler gradient (Eppendorf, Hamburg, Germany), with the following program: initial denaturation for 3 min at 94°C, 37 cycles of denaturation for 45 s at 94°C; 54°C; and 56°C annealing temperatures for 18 S rDNA and 28 S rDNA, respectively; extension for 45 s to 1 min at 72°C, and finally an extension step of 6 min at 72°C followed by a temperature on hold at 4°C. After DNA amplification, 4 µL of product from each tube was loaded on a 1% agarose gel in TBE buffer (40 mM Tris, 40 mM boric acid, and 1 mM EDTA) for evaluation of the DNA bands. The bands were stained with ethidium bromide and visualized and photographed on a UV transilluminator. The amplicons of each gene were stored at –20°C. Finally, the PCR products were purified for sequencing by Inqaba Biotech (South Africa). Available sequences for other *Bitylenchus* spp. were obtained from NCBI GenBank for comparison. Also, as outgroups, *Coslenchus costatus* (De Man, 1921) Siddiqi, 1978 (KX156285; DQ328719) based on Handoo et al. (2014) were used as the outgroup for the 18 S and 28 S rDNA analyses, respectively. The ribosomal DNA sequences were analyzed and edited with BioEdit (Hall, 1999) and or aligned using CLUSTAL W (Thompson et al., 1994). The length of the alignments was 1,772 and 820 bps for 18 and 28 S rDNA, respectively. Phylogenetic trees were generated using the Bayesian inference method as implemented in the program Mr. Bayes 3.1.2 (Ronquist and Huelsenbeck, 2003). The GTR+I+G model was selected using jModeltest 2.1.10 (Guindon and Gascuel, 2003; Darriba et al., 2012). Then, the chosen model was initiated with a random starting tree and run with the Markov chain Monte Carlo (MCMC) for 106 generations. The trees visualized using TreeView ver. 1 (Page, 2002). The original partial 18 S rDNA and 28 S (D2-D3 expansion) sequences of *B. ventrosignatus* were deposited in GenBank under the accession numbers MW255611 (18 S rDNA) and MW255612–MW255613 (28 S rDNA), respectively.

## Results

### *Bitylenchus ventrosignatus* (Tobar Jiménez, 1969) Siddiqi, 1986

**Figure 1:**
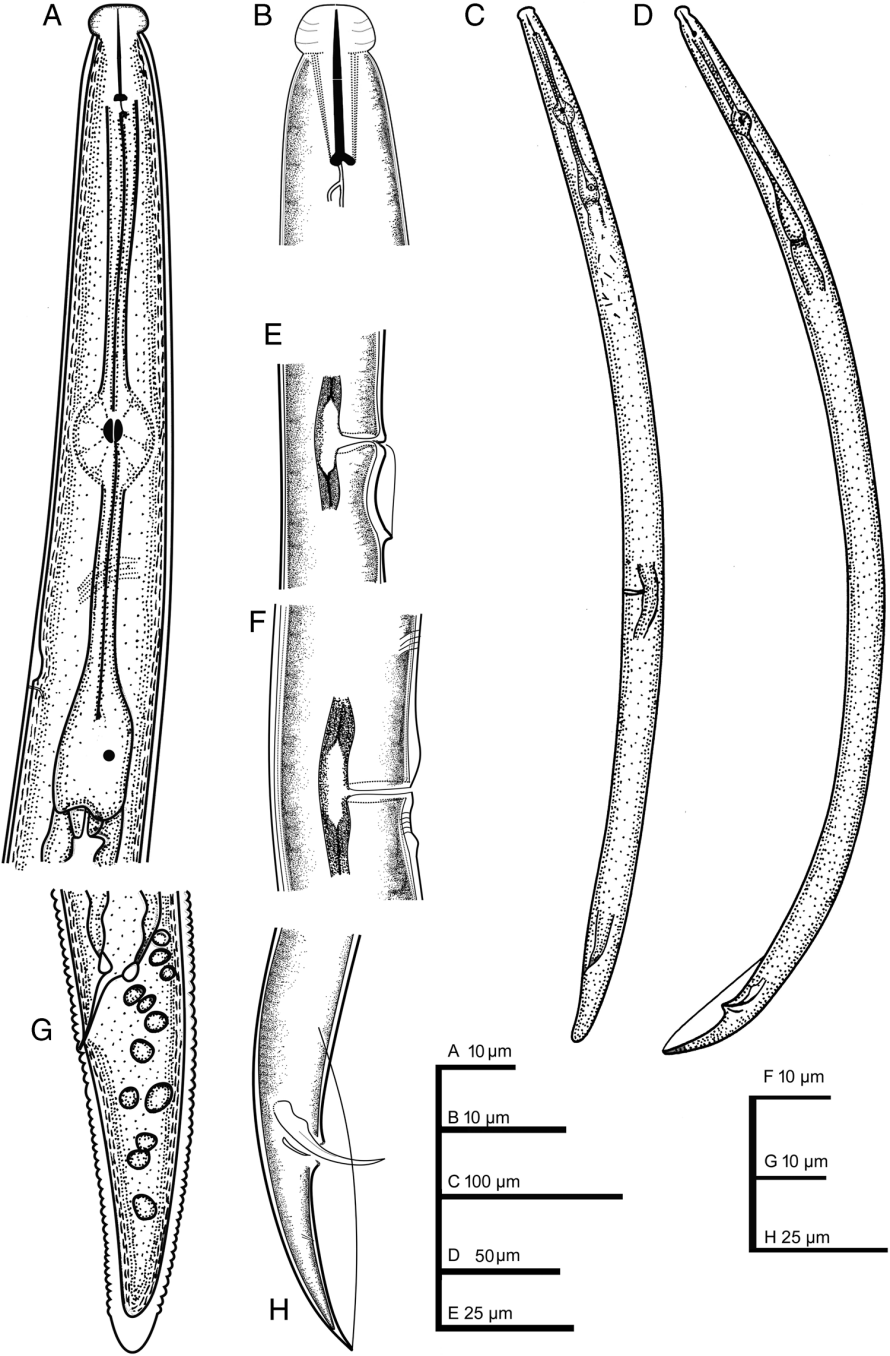
*Bitylenchus ventrosignatus* (Tobar Jiménez, 1969) Siddiqi, 1986. (A) Anterior end; (B) Anterior end (stylet and dgo); (C) Entire female; (D) Entire male; (E-F) Vaginal irregular undulation; (G) Female posterior end; (H) Male posterior end.

**Figure 2:**
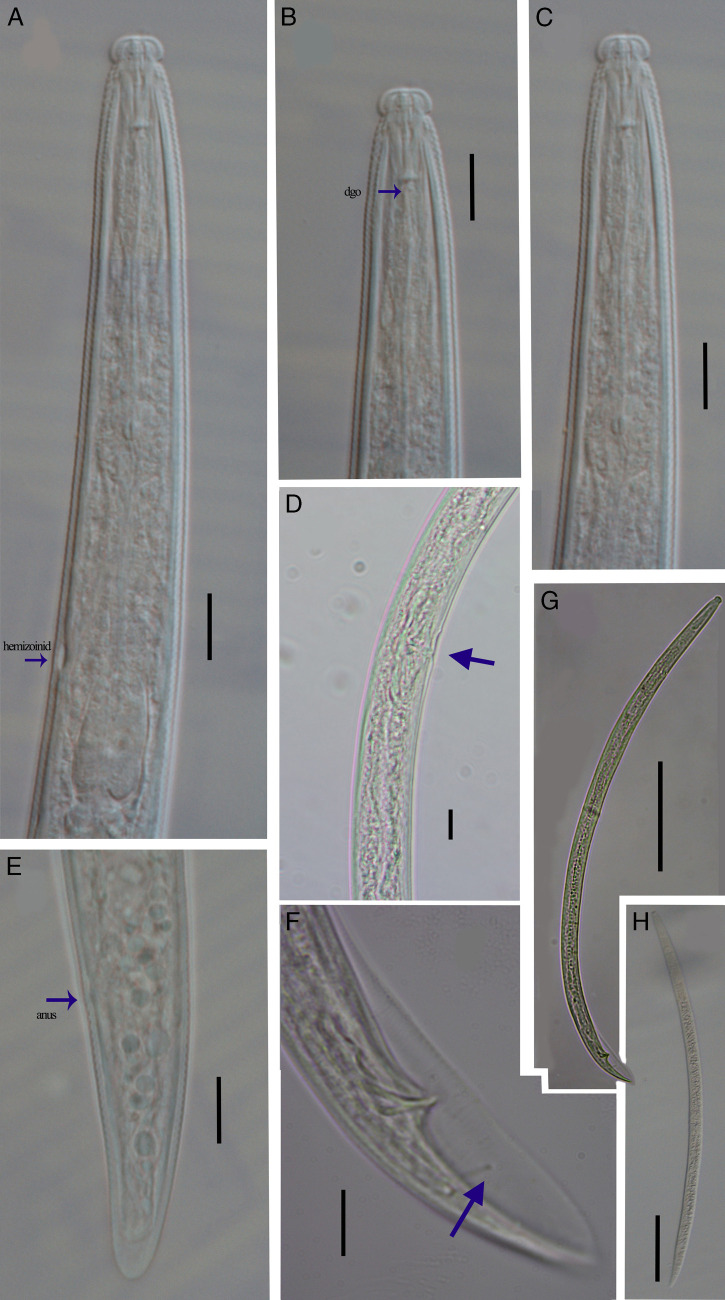
*Bitylenchus ventrosignatus* (Tobar Jiménez, 1969) Siddiqi, 1986. (A) Anterior end (arrow indicates hemizonid); (B) Anterior end (arrow indicates dgo); (C) Anterior end (stoma and median bulb); (D) Vagina region (arrow indicates undulation); (E) Female posterior end (arrow indicates anus); (F) Male posterior end (arrow indicates phasmid); (G) Entire male; (H) Entire female (Scale bar: 10 µm; except for G, H 100 µm).

(Figs. 1 and 2; Table 1).

**Table 1.** Measurements of females and males of *B. ventrosignatus* from Botswana.

n	5 ♀♀	5 ♂♂
L	526.7 ± 38.8 (496 – 583)	498 ± 23.3 (470 – 521)
a	25.3 ± 1.1 (23.7–26.3)	29.9 ± 3.4 (28.0 – 34.3)
b	4.8 ± 0.2 (4.6 – 5.1)	4.5 ± 0.1 (4.4 – 4.7)
c	13.2 ± 1.4 (11.5 –14.2)	13.7 ± 1.2 (12.1–14.8)
c'	2.9 ± 0.4 (2.7– 3.4)	3.2 ± 0.1 (3.1– 3.5)
V	55.8 ± 0.4 (55 – 56)	–
Lip region height	3.8 ± 0.3 (3 – 4)	4.2 ± 0.4 (3.7– 4.6)
Lip region diameter	7.4 ± 0.8 (6–8)	6.8 ± 0.7 (6.2–7.7)
Stylet	13.5 ± 0.6 (13 –14)	14 ± 0.2 (13.7–14.4)
m	39.5 ± 26.3 (51– 54)	52.1 ± 10.1 (40 – 63)
Median bulb to anterior end	52.3 ± 3.3 (48 – 56)	54.1 ± 3.4 (49 – 56)
MB	50.1 ± 1.6 (47– 51)	50.2 ± 0.1 (50 – 51)
Excretory pore to anterior end	87.7 ± 4.2 (85 – 94)	81.6 ± 4.2 (79 – 88)
Pharynx	96.3 ± 4.5 (92–101)	97 ± 0.4 (96 – 97)
Neck	107.6 ± 5.4 (102–114)	111.1 ± 0.3 (110 –112)
Neck base diameter	18.4 ± 2.4 (16 – 21)	15.9 ± 1.2 (14.5 –17.0)
Mid-body diameter	20.8 ± 1.9 (19 –23)	16.7 ± 1.1 (15 –18)
Anal body diameter	14.1 ± 1.3 (13 –16)	11.2 ± 0.1 (11.0 –11.4)
Lateral filed width	4.3 ± 1.1 (3– 6)	4.2 ± 0.1 (4.2– 4.3)
Vulva anterior end	294.5 ± 23.9 (275 – 329)	–
Anus anterior end	489 ± 45.1 (461– 541)	461.6 ± 25.0 (431– 478)
Tail length	40.3 ± 4.7 (35 – 44)	36.3 ± 1.8 (35 – 39)
Phasmid	15.1 ± 3.5 (11–18)	13.3 ± 0.6 (12.7–14.2)
Tail annuli	30 ± 6.2 (30 – 35)	
Spicules	–	22.6 ± 1.2 (21–24)
Gubernaculum	–	8.3 ± 0.4 (8 –9)
Bursa length	–	67.1 ± 7.9 (59 –76)

Female (*n* = 5): Body almost open C-shaped after heat relaxation, no longitudinal striae or ridges outside lateral fields. Body annuli distinct but fine, 0.8–1.2 μm wide around mid-body. Lateral fields originating at the level of the conus of the stylet and extending up to hyaline region of tail to tail terminus, with four incisures, 13–26% of the corresponding body diameter. Lip region high, spherical, offset to body contour, 3.8 ± 0.3 (3–4) μm height, 7.4 ± 0.8 (6–8) μm diameter; with four annuli. Stoma comprises cheilostom (=conus) 52–54% of the stoma length, gymnostom (=almost part of the shaft) 38–40% of the stoma length, and prostegostom (=posterior part of the shaft and knobs) 8–9% of the stoma length. Stylet moderately strong, conus slightly longer than shafte; knobs laterally to posteriorly directed. Dorsal gland orifice about 1.4–2.5 μm long behind stylet base. Median pharyngeal bulb rounded; basal bulb pyriform. Cardia well developed. Hemizonid usually just two to three annuli anterior to excretory pore, 1.0–1.5 annuli wide. Vulva a transverse slit slightly posterior to the middle of the body, vagina with 9.3 ± 1.6 (7.3–11.4) µm length. Epiptygma absent. Cuticle posterior to vulva with undulation. Reproductive system amphidelphic, didelphic; anterior (one measurement, 122 µm) and posterior (one measurement, 126 µm) ovaries well developed. Spermatheca rounded, filled with rounded spermatozoa. Tail subcylindrical, tail terminus rounded or conical and smooth. Phasmids located slightly anterior to middle of the tail, 38–42% of tail length. Post-anal intestinal sac present.

Male (*n*=5): Body J-shaped after relaxation. Abundant, similar to the females morphologically, except for the reproductive system. Testis one, outstretched anteriorly. Spicules tylenchoid, paired and symmetrical, 8–10 times longer than wide: slightly elongate and ventrally curved, rounded manubrium, short and straight calamus, and ventrad curved lamina with an acute tip, bursa large and conspicuous, extending to tail tip, 59–76 µm long. Gubernaculum are well developed, curved, about 34–41% of the spicule length. Tail terminus conoid-pointed.

**Figure 3:**
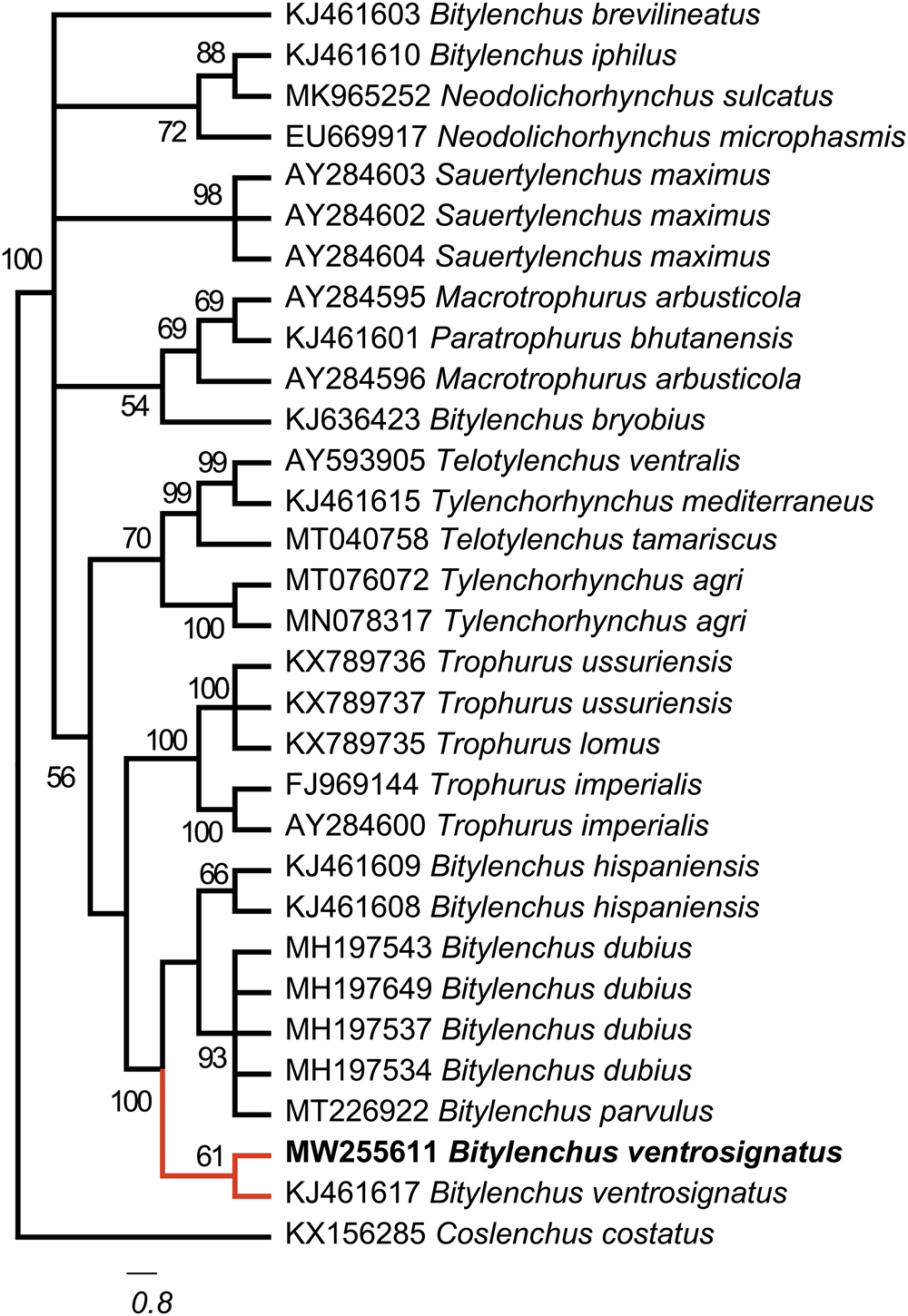
The Bayesian tree inferred from known and newly sequenced *Bitylenchus ventrosignatus* from Botswana based on the 18 S rDNA region.

**Figure 4:**
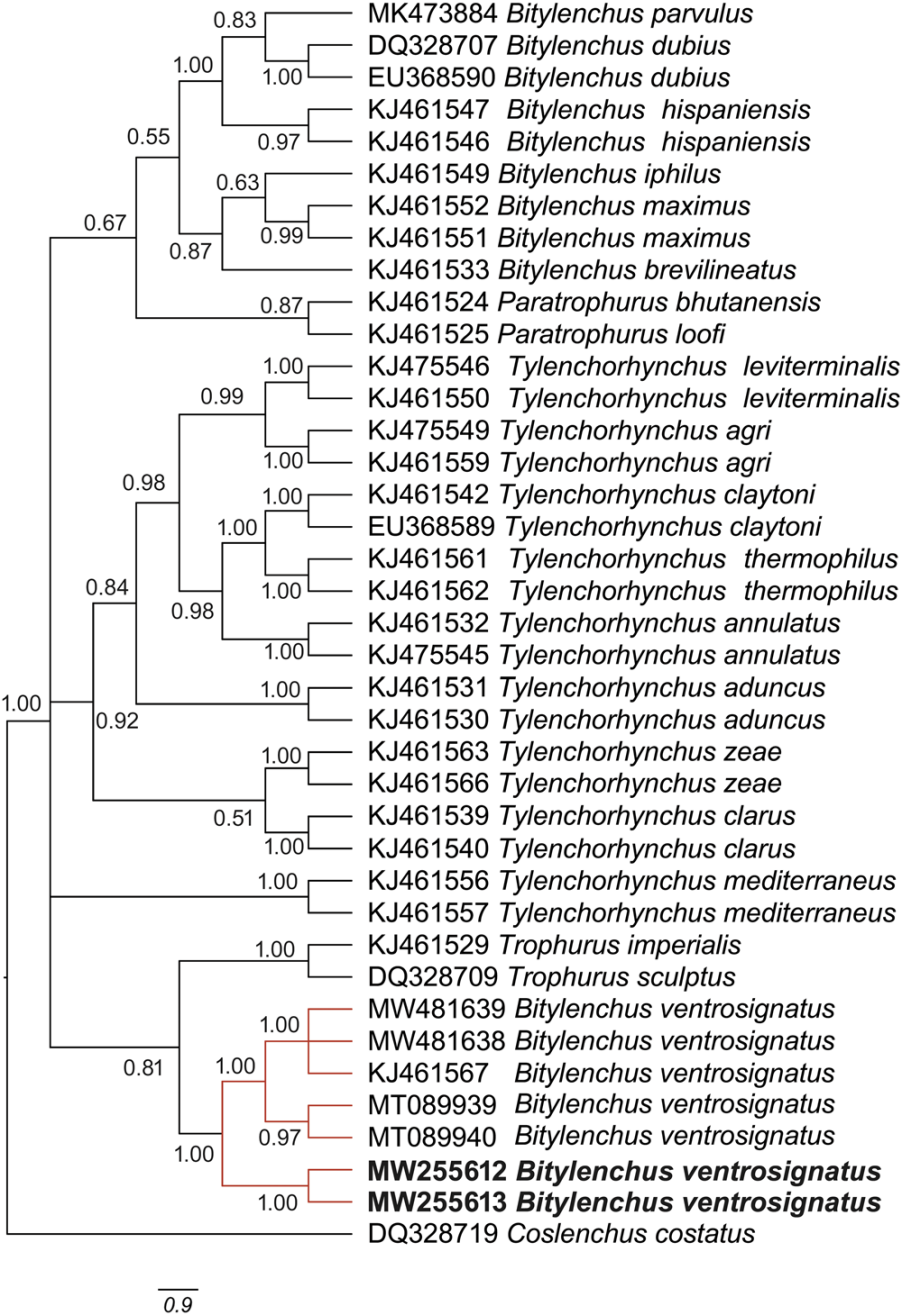
The Bayesian tree inferred from known and newly sequenced *Bitylenchus ventrosignatus* from Botswana based on the 28 S rDNA region.

Phylogenetic analysis The Bayesian inference tree of 18 S rDNA of *Bitylenchus* species (Fig. 3) placed the Botswanan *B. ventrosignatus* close to Spanish *B. ventrosignatus* (acc. nr: KJ461617) with 0.61 posterior probability. In contrast, the Bayesian tree of 28 S rDNA (Fig. 4), placed Botswanan *B. ventrosignatus* close to the Spanish (KJ461567), Iranian (MW481638; MW481639) and Tanzanian (MT089939; MT089940) populations of *B. ventrosignatus* with 1.00 posterior probability.

## Discussion

Overall, the morphology and morphometrics are in agreement with those reported by Fortuner and Luc (1987) and Geraert (2011). However, Fortuner and Luc (1987) reported *B. ventrosignatus* lacks a postanal intestinal sac, the character observed in the Botswanan specimens (Fig. 2E). Compared with the material examined by Handoo et al. (2014), specimens from Botswana have smaller female body length (496–583 vs 610–722 µm), female tail length (35–44 vs 41–50 µm), fewer tail annuli (30–35 vs 32–42) and smaller gubernaculum (8–9 vs 10–12 µm). Compared with the material examined by Geraert et al. (1975), they differ in the female stylet length (13–14 vs 12.5–15 µm), female neck length (102–114 vs 98–121 µm), and male body length (470–521 vs 560–580 µm). In addition, the cuticle around the vulva showed ventral line irregular undulations (Figs. 1E-F and 2D). This character has been described in the original description for *B. ventrosignatus* (Geraert et al., 1975; Geraert, 2011; Tobar Jiménez, 1969). Irregular line undulation has been described for *B. parvulus* Hosseinvand et al., 2020; however, they differ with the tested species in body length (496–583 vs 542–834 µm), stylet length (13–14 vs 17–18.5 µm), and tail length (35–44 vs 42–59 µm).

The lateral field also areolated; the character has been reported by Handoo et al. (2014) for this species. Despite morphological similarities with *B. zambiensis* (Venditti and Noel, 1995) Siddiqi, 2000, they differ in tail length (35–44 vs 35–56 µm), tail annuli (30–35 vs 21–32), spicule length (21–24 vs 17–22 µm), and gubernaculum length (8–9 vs 9–12 µm). In addition, they differ in the vulval region (posterior with irregular undulation vs lacking irregular undulation). However, compared with *T. fatimae* Khan et al., 2004, they differ in the basal bulb (pyriform vs cylindrical), gubernaculum length (8–9 vs 11.5–12 µm), and irregular undulation at the posterior part of the vulva (present vs absent) (see Geraert, 2011). Morphometrical differences of the Botswanan population compared with the other populations of the same species are considered a geographical distribution, and therefore, the present species identified as *B. ventrosignatus*. Besides, the 18 S and 28 S rDNA markers confirmed this species as *B. ventrosignatus*. The sequence lengths of the 18 S rDNA and 28 S region of *B. ventrosignatus* isolate are 859 and 711 base pairs long, respectively. The nBlast comparison of 18 S rDNA showed that the test population has 98% similarity to the Spanish population of *B. ventrosignatus* (KJ461617). In contrast, the 28 S rDNA showed 95% similarity of the Botswanan and Spanish population (KJ461567) of *B. ventrosignatus*. In addition, 28 S rDNA marker indicated Botswanan *B. ventrosignatus* has 95 and 96% similarity with Tanzanian (MT089939; MT089940) and Iranian (MW481638; MW481639) populations of *B. ventrosignatus*, respectively. Despite the high similarity of the studied species and *B. ventrosignatus*, the other species and populations of *Bitylenchus* showed the lowest similarity. The results showed 87% similarity to *B. iphilus* (KJ461549) for the 28 S rDNA and 94% similarity to *B. bryobius* (KJ636423) for the 18 S rDNA marker.

The phylogenetic analysis using 18 S and 28 S rDNA, placed the Botswanan *B. ventrosignatus* in a clade together with other *B. ventrosignatus* populations (Figs. 3 and 4). The phylogenetic analysis of *B. ventrosignatus* placed these populations at the base of the phylogenetic trees. This topology would be consistent with suggesting that the species may represent a separate genus as suggested in Handoo et al. (2014). With the inclusion of *B. ventrosignatus*, the phylogenetic analysis demonstrated that the genus *Bitylenchus* is not a monophyletic group. This is in agreement with Handoo et al. (2014). Besides, the results obtained by Hosseinvand et al. (2020) indicated that *Bitylenchus* species divide into two groups. However, more sequences should be included aiming for the phylogenetic study, albeit the species identification of the genus *Bitylenchus* is problematic.

Furthermore, the SEM study and mtDNA (e.g., COI) may reveal the species’ real position belongs to *Bitylenchus*. Overall, the current study’s findings were in agreement with other *Bitylenchus* 18 S and 28 S rDNA phylogenies (Handoo et al., 2014; Hosseinvand et al., 2020). Two permanent microscope slides containing the five females and five males were deposited in the Nematology collection of the University of Limpopo, South Africa. Relative to published literature, this is the first record of *B. ventrosignatus* from natural areas of Botswana.

## References

[ref001] Abolafia, J. and Peña-Santiago, R. 2017. On the identity of *Chiloplacus magnus* Rashid & Heyns, 1990 and *C. insularis* Orselli & Vinciguerra, 2002 (Rhabditida: Cephalobidae), two confusable species. Nematology 19:1017–1034, doi: 10.1163/15685411-00003104.

[ref002] Baermann, G. 1917. Eine einfache Methode zur Auffindung von Ankylostomum (Nematoden) Larven in Erdproben. Geneeskunding Tijdschrift voor Nederlandsch-Indië 57:131–137.

[ref003] Baldwin, J. G., Bumbarger, D. and Ragsdale, E. 2004. Revised hypotheses for phylogenetic homology of the stomatostylet in tylenchid nematodes. Nematology 6:623–632, available at: 10.1163/1568541042843559.

[ref004] Blaxter, M. L., De Ley, P., Garey, G. R., Liu, L. X., Scheldeman, P., Vierstraete, A., Vanfleteren, J. R., Mackey, L. Y., Dorris, M., Frisse, L. M., Vida, J. T. and Thomas, W. K. 1998. A molecular evolutionary framework for the phylum Nematoda. Nature 392:71–75. 9510248 10.1038/32160

[ref005] Chitwood, B. G. 1950. “An outline classification of the nematodes”, In Chitwood, B. G. and Chitwood, M. B. (Eds), An Introduction to Nematology. I. Anatomy Monumental Printing Co., Baltimore, MD, pp. 12–27.

[ref006] Darriba, D., Taboada, G. L., Doallo, R. and Posada, D. 2012. jModelTest 2: more models, new heuristics and parallel computing. Nature Methods 9:772, available at: 10.1038/nmeth.2109. PMC4594756 22847109

[ref007] De Grisse, A. 1969. Redescription ou modifications de quelques techniques utililisés dans l’étude des nématodes phytoparasitaires. Mededelingen van de Rijksfaculteit Landbouwetenschappen Gent 34:351–369.

[ref008] De Ley, P., Felix, M. A., Frisse, L. M., Nadler, S. A., Sternberg, P. W. and Thomas, W. K. 1999. Molecular and morphological characterisation of two reproductively isolated species with mirror-image anatomy (Nematoda: Cephalobidae). Nematology 2:591–612, available at: 10.1163/156854199508559.

[ref009] De Man, J. G. 1880. Die einheimischen, frei in der reinen Erde und im süssen Wasser lebenden Nematoden. Vorläufiger Bericht und descriptiv-systematischer Theil. Tijdschrift nederlandsche Dierkundige Vereeniging 5:1–104.

[ref010] De Man, J. G. 1921. Nouvelles recherches sur les nematodes terricoles de la Hollande. Capita Zoologica 1:3–62.

[ref011] Fortuner, R. and Luc, M. 1987. A reappraisal of Tylenchina (Nemata). 6. The family Belonolaimidae Whitehead, 1960. Revue de Nématologie 10:183–202.

[ref013] Geraert, E. 2011. The Dolichodoridae of the world, identification of the family Dolichodoridae (Nematoda: Tylenchida), Academia Press, Ghent, p. 520.

[ref012] Geraert, E., Zepp, A. and Borazanci, N. 1975. Some plant nematodes from Turkey. Mededelingen van de Faculteit Landbouwwetenschappen, Rijksuniversteit Gent 40:511–515.

[ref014] Guindon, S. and Gascuel, O. 2003. A simple, fast and accurate method to estimate large phylogenies by maximum-likelihood. Systematic Biology 52:696–704, available at: 10.1080/10635150390235520 14530136

[ref015] Hall, T. A. 1999. BioEdit: a user-friendly biological sequence alignment editor and analysis program for Windows 95/98/NT. Nucleic Acids Symposium Series 41:95–98.

[ref016] Handoo, Z. A., Palomares-Rius, J. E., Cantalapiedra-Navarrete, C., Liébanas, G., Subbotin, S. A. and Castillo, P. 2014. Integrative taxonomy of the stunt nematodes of the genera *Bitylenchus* and *Tylenchorhynchus* (Nematoda, Telotylenchidae) with description of two new species and a molecular phylogeny. Zoological Journal of the Linnean Society 172:231–264, available at: 10.1111/zoj12175

[ref017] Hosseinvand, M., Eskandari, A., Ganjkhanloo, S., Ghaderi, R., Castillo, P. and Palomares-Rius, J. E. 2020. Taxonomical considerations and molecular phylogeny of the closely related genera *Bitylenchus*, *Sauertylenchus* and *Tylenchorhynchus* (Nematoda: Telotylenchinae), with one new and four known species from Iran. Journal of Helminthology 94:1–25, available at: 10.1017/S0022149X20000784. 32998785

[ref018] Khan, H. A., Saeed, H. and Akhter, M. 2004. A new species of *Tylenchorhynchus* with comments on *Geocenamus rugosus* (Thorne and Malek, 1968) Brzeski, 1991 from Sindh. Pakistan Journal of Scientific and Industrial Research 47:446–450.

[ref019] Page, R. D. M. 2002. “Visualizing phylogenetic trees using TreeView”, Curr Protoc Bioinformatics Chapter 6: Unit 6.2, Wiley Online Library, available at: 10.1002/0471250953.bi0602s01 18792942

[ref020] Ronquist, F. and Huelsenbeck, J. 2003. MrBayes 3: Bayesian phylogenetic inference under mixed models. Bioinformatics 19:1572–1574, available at: 10.1093/bioinformatics/btg180 12912839

[ref021] Shokoohi, E., Abolafia, J. and Mashela, P. W. 2020. Redescription of *Paratrophurus anomalus* from South Africa. Nematology 22:543–554, available at: 10.1163/15685411-00003322.

[ref022] Siddiqi, M. R. 1978. The unusual position of the phasmids in *Coslenchus costatus* (De Man, 1921) gen. n., comb. n. and other Tylenchidae (Nematoda: Tylenchida). Nematologica 24:449–455, available at: 10.1163/187529278X00597.

[ref023] Siddiqi, M. R. 1986. Tylenchida: parasites of plants and insects, Commonwealth Agricultural Bureaux, Farnham Royal, London, p. 645

[ref024] Siddiqi, M. R. 2000. Tylenchida: parasites of plants and insects. 2nd ed., CABI Publishing, Wallingford, p. 833

[ref025] Straube, D. and Juen, A. 2013. Storage and shipping of tissue samples for DNA analyses: a case study on earthworms. European Journal of Soil Biology 57:13–18, available at: 10.1016/j.ejsobi.2013.04.001. 26109838 PMC4461180

[ref026] Thompson, J. D., Higgins, D. G. and Gibson, T. J. 1994. CLUSTAL W: improving the sensitivity of progressive multiple sequence alignment through sequence weighting, position-specific gap penalties and weight matrix choice. Nucleic Acids Research 22:4673–4680. 7984417 10.1093/nar/22.22.4673 PMC308517

[ref027] Tobar Jiménez, A. 1969. Descripción del *Tylenchorhynchus ventrosignatus* sp. nov. (Nematoda: Tylenchida). Revista Ibérica de Parasitologia 29:399–403.

[ref028] Venditti, M. E. and Noel, G. R. 1995. Description of *Tylenchorhynchus zambiensis* n. sp. (Nemata: Tylenchidae) from Zambia. Nematropica 25:1–6.

